# Fully Inkjet-Printed Flexible Graphene–Prussian Blue Platform for Electrochemical Biosensing

**DOI:** 10.3390/bios15010028

**Published:** 2025-01-08

**Authors:** Željka Boček, Marko Zubak, Petar Kassal

**Affiliations:** University of Zagreb, Faculty of Chemical Engineering & Technology, Trg Marka Marulića 19, 10000 Zagreb, Croatia; zbocek@fkit.unizg.hr (Ž.B.); mzubak@fkit.unizg.hr (M.Z.)

**Keywords:** Prussian Blue, inkjet printing, flexible biosensor, intense pulsed light, enzymatic sensor, lactate sensor, sweat lactate

## Abstract

Prussian Blue (PB) is commonly incorporated into screen-printed enzymatic devices since it enables the determination of the enzymatically produced hydrogen peroxide at low potentials. Inkjet printing is gaining popularity in the development of electrochemical sensors as a substitute for screen printing. This work presents a fully inkjet-printed graphene–Prussian Blue platform, which can be paired with oxidase enzymes to prepare a biosensor of choice. The graphene electrode was inkjet-printed on a flexible polyimide substrate and then thermally and photonically treated with intense pulsed light, followed by inkjet printing of a PB nanoparticle suspension. The optimization of post-printing treatment and electrode deposition conditions was performed to yield a platform with minimal sheet resistance and peak potential differences. A thorough study of PB deposition was conducted: the fully inkjet-printed system was compared against sensors with PB deposited chemically or by drop casting the PB suspension on different kinds of carbon electrodes (glassy carbon, commercial screen-printed, and in-house inkjet-printed electrodes). For hydrogen peroxide detection, the fully inkjet-printed platform exhibits excellent sensitivity, a wider linear range, better linearity, and greater stability towards higher concentrations of peroxide than the other tested electrodes. Finally, lactate oxidase was immobilized in a chitosan matrix, and the prepared biosensor exhibited analytical performance comparable to other lactate sensors found in the literature in a wide, physiologically relevant linear range for measuring lactate concentration in sweat. The development of mediator-modified electrodes with a single fabrication technology, as demonstrated here, paves the way for the scalable production of low-cost, wearable, and flexible biosensors.

## 1. Introduction

Prussian Blue’s property to selectively oxidize hydrogen peroxide at low potentials (~0 V vs. Ag/AgCl), as well as its relative ease and low cost of production, makes it an attractive material for biosensor assembly, especially for oxidase-based biosensors. Low operating potential improves the selectivity of the biosensor by eliminating the possible interferences present in physiological fluids (such as ascorbic acid and uric acid), which would risk oxidation at potentials usually required for direct hydrogen peroxide oxidation (~0.7 V vs. Ag/AgCl) [1]. So far, Prussian Blue has been successfully paired with various oxidase enzymes, such as glucose oxidase [2,3], cholesterol oxidase [4], choline oxidase [5], urate oxidase [6], and lactate oxidase [3,7,8,9,10,11]. Its inherent flaw lies in its instability in neutral and alkaline media, as well as its inevitable decomposition when employed as an H_2_O_2_ reduction catalyst since this reduction itself produces hydroxide ions [12,13]. However, this flaw can be tolerated in favor of using it for disposable, one-time-use biosensors.

Reproducible and scalable fabrication technologies are needed to achieve distributed electrochemical sensors [14]. Screen-printing is a well-established, reliable and inexpensive fabrication method that has been extensively used for biosensor fabrication. It offers simplicity and high reproducibility for the production of disposable biosensing platforms. Conductive carbon pastes have commonly been combined with Prussian Blue to realize screen-printed electrodes paired with oxidase enzymes [6,10,15,16,17]. However, while it has been commercialized and employed for the production of commercially available biosensing solutions, every change to the biosensor platform design requires the production of new physical screens, which impedes prototyping. Moreover, the use of thick inks prevents the use of solutions or solubilized materials, as well as being unsuitable for sensitive materials or substrates that would not handle the contact nature of this production method [18].

Inkjet printing is a completely digital, non-contact technique that does not require the use of any additional stencils when it comes to designing and printing a new pattern since the process is fully computerized and only requires digital input. It offers faster, more cost-effective, and less wasteful mass fabrication of sensors with better resolution compared to more traditional methods [19,20]. Moreover, the printing process itself is based on using solubilized inks, unlike the paste-like inks used for screen printing that require contact with the substrate. This makes inkjet printing suitable both for sensitive substrates and sensitive materials that need to be printed. Inkjet printing has therefore already been used in the development of electrochemical hydrogen peroxide sensors [21,22,23,24,25,26]. Nevertheless, fully inkjet-printed systems have reduced linear ranges and lower sensitivities compared to screen-printed sensors. Inkjet printing is therefore currently more often used as a complimentary deposition method to screen printing in the fabrication of electrochemical sensors as a way of reproducibly replacing the dominant drop-casting method [19,27].

Although the deposition of less material is advantageous from the standpoint of cost and waste reduction, this also means that inkjet-printed electrodes are less conductive than thicker, screen-printed electrodes (which has a detrimental effect on analytical parameters). This problem becomes more pronounced with the inclusion of a non-conducting material, such as Prussian Blue. As the inks often require polymeric stabilizers to keep the functional nanoparticle ink material from agglomeration, conductivity is also a common issue with the final printed product. Stabilizers are most often removed by thermal processing, yet such processing is time-consuming, and its use limits the inkjet printing application to thermally stable substrates. An alternative photonic treatment approach via intense pulsed light (IPL) flashing can rapidly heat up the printed films, decomposing the stabilizers and leaving the thermally sensitive substrate undamaged [28]. This is especially true for graphene-based inks, as the photothermal sintering occurs selectively and rapidly on the printed graphene film, thanks to the difference in optical absorption properties between the graphene and the substrate it is printed on [29]. Moreover, photonic processing is compatible with rapid mass production methods since it is easily integrated into in-line production [30].

In the development of disposable biosensors, Prussian Blue is commonly deposited on conductive substrates (e.g., printed electrodes) using a chemical deposition method via drop casting [2,7], which negatively affects reproducibility. To alleviate this, several examples exist where a Prussian Blue nanoparticle suspension has been inkjet-printed onto electrodes obtained using other technologies [4,27,31,32]. Nevertheless, fabricating electrodes for biosensors with two different printing techniques (e.g., screen printing and inkjet printing) requires additional equipment and significantly increases cost.

In this work, we introduce a fully inkjet-printed biosensor platform based on Prussian Blue as a mediator. To the best of our knowledge, this is the first time both the electrode material (in this case graphene) and Prussian Blue have been fully inkjet-printed for assembling a complete hydrogen peroxide-sensitive platform, which is suitable for further modification with enzymes to produce a biosensor of choice. The post-print processing of the inkjet-printed graphene electrodes via IPL was optimized to improve electrical and electrochemical properties. For comparison, we also employed a chemical deposition method described previously [2], and both methods were evaluated on commercial glassy carbon electrodes (GCEs), screen-printed carbon electrodes (SPEs), and finally, on our inkjet-printed (IJP) graphene electrodes. Finally, as a proof of concept, a lactate biosensor was assembled by immobilizing lactate oxidase (LOx) with the use of chitosan and poly(vinyl) chloride to test out a biosensor with simplicity in mind.

## 2. Materials and Methods

### 2.1. Chemicals and Materials

Potassium ferricyanide (K_3_[Fe(CN)_6_]) and potassium dihydrogen phosphate (KH_2_PO_4_) were purchased from Merck, Darmstadt, Germany. Potassium ferrocyanide trihydrate (K_4_[Fe(CN)_6_] × 3 H_2_O) and potassium chloride (KCl) were purchased from Kemika, Zagreb, Croatia. Iron(III) chloride hexahydrate (FeCl_3_ × 6 H_2_O), potassium hydrogen phosphate (K_2_HPO_4_), and hydrogen peroxide (H_2_O_2_), 30% wt., were purchased from Gram-Mol, Zagreb, Croatia. Hydrochloric acid, 35% wt., was purchased from Lach-Ner, Neratovice, Czech Republic. Graphene ink (900695), lactate oxidase from *Aerococcus viridans*, L-(+)-lactic acid, chitosan (medium molecular weight), and tetrahydrofuran (THF, 99.5% wt.) were purchased from Sigma Aldrich, Burlington, MA, USA. Acetic acid, 99.5% wt., was purchased from T.T.T., Sveta Nedelja, Croatia. Polyvinyl chloride (PVC, high molecular weight) was obtained from Fluka, Buchs, Switzerland. All chemicals were pro-analysis grade and were used as received. All solutions were prepared using double-distilled deionized water (MilliQ, Millipore, Burlington, MA, USA). Polyimide (PI) sheets were purchased from DuPont, Wilmington, DE, USA. A commercial glassy carbon electrode was obtained from BASi, West Lafayette, IN, USA. Commercial screen-printed carbon electrodes (ED-S1PE-C10) were purchased from MicruX Technologies, Gijón, Spain. A single junction Ag/AgCl/3M NaCl reference electrode was purchased from BASi, West Lafayette, IN, USA. A double junction Ag/AgCl/3M KCl/3M KCl electrode was obtained from Metrohm, Herisau, Switzerland.

### 2.2. Inkjet Printing, Post-Print Treatment, and Characterization of Graphene Electrodes

**Figure 1:**
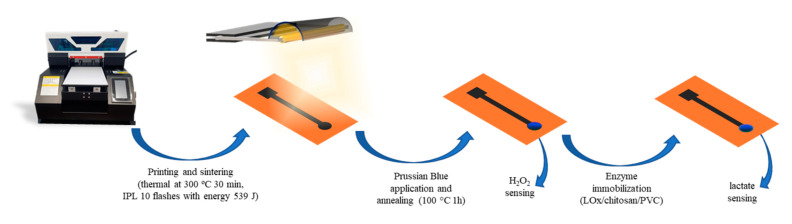
Schematic representation of the inkjet-printed electrode assembly and modification.

Inkjet-printed graphene electrodes were printed with graphene ink on a Gateway inkjet printer with an Epson L800 printhead. The entire planar electrode body (including the contact strip) was printed with 3 layers of graphene ink on a polyimide sheet. After printing, the electrode underwent thermal (300 °C for 30 min) and photothermal treatment (IPL flashing with Xenon X1100 system), rendering the substrate conductive (Figure 1). Different numbers of flashes at 539 J were tested during optimization. Four point probe resistance measurements were conducted with Ossila Four Point Probe. FTIR spectra were obtained using an IRTracer-100 spectrometer (Shimadzu). When necessary, PB-IJPs were characterized with an added chitosan layer, applied by drop casting three aliquots of 3 µL of chitosan solution (1 wt% in 0.1 M HAc).

### 2.3. Synthesis and Characterization of Prussian Blue

#### 2.3.1. Chemical Synthesis

The chemical deposition of Prussian Blue (PB) was conducted according to previous work by Ricci et al. [2]. A solution of 0.1 M K_3_[Fe(CN)_6_] in 0.1 M KCl and 0.01 M HCl, as well as a solution of 0.1 M FeCl_3_ in 0.1 M KCl and 0.01 M HCl, were freshly prepared and briefly mixed in equimolar amount. An amount of 40 µL of the reaction mixture was drop cast on the working electrode area (*d* = 3 mm). The synthesis of Prussian Blue was conducted for 10, 20, or 60 min. After deposition, the reaction mixture was aspirated, and the electrodes were rinsed with 0.01 M HCl solution. The resulting PB-modified electrodes were dried at 100 °C for 1 h. When needed, the working area of the printed electrodes was limited by punching a hole in the self-adhesive foil and carefully applied to expose only the working area for testing.

#### 2.3.2. Prussian Blue Nanoparticle Suspension

The Prussian Blue nanoparticle suspension, later denoted in this work as PBNP suspension, was prepared according to previous work [27,31]. An amount of 1 mL of 0.1 M KCl and a 0.01 M HCl solution was added to a vial, followed by 2 mL of 2 mM K_4_[Fe(CN)_6_]. Next, 2 mL of 2 mM FeCl_3_ was added dropwise to the mixture with vigorous stirring. The mixture gradually turns blue, indicating the formation of Prussian Blue suspension. The mixture was stirred overnight to ensure the reaction was complete.

The suspension was investigated after synthesis with UV/Vis measurements for two hours to assess the stability of the suspension (100 times diluted in PBNP matrix solution containing 20 mM KCl and 2 mM HCl). Visually, the suspension remained stable and without precipitation for at least a week after synthesis.

Dynamic light scattering (DLS) and electrophoretic light scattering (ELS) measurements were conducted on Malvern Zetasizer Ultra (Malvern Panalytical Ltd., Malvern, UK). Dynamic light scattering (DLS) was conducted by diluting the suspension 100 times in three different media: −1 mM HCl, 1 mM NaOH, and pure deionized water. Electrophoretic light scattering (ELS) was conducted by diluting the suspension in pure deionized water 100 times to determine the *ς*-potential of the suspension.

The suspension was used to modify the electrodes either by drop casting or printing a 4 × 4 mm square over the working electrode area (*d* = 3 mm). Printing of the PBNP suspension was conducted on Dimatix Materials Printer DMP-2850 with a drop spacing of 15 µm, using the Samba cartridge with all 12 nozzles engaged. The printing of PBNP suspension was conducted only on inkjet-printed electrodes, followed by the usual drying step at 100 °C for 1 h.

### 2.4. Electrochemical Measurements

Electrochemical characterization was conducted via cyclic voltammetry on an EG&G Princeton Applied Research Model 263A potentiostat (Oak Ridge, TN, USA) in a three-electrode system comprising a single junction Ag/AgCl/3M NaCl reference electrode and a platinum wire as a counter electrode. The working electrode was a bare or PB-modified glassy carbon electrode (GCE), screen-printed carbon electrode (SPE), or inkjet-printed electrode (IJP). Unless otherwise specified, the measurements were taken by cycling the potential between −0.2 and +0.5 V at 50 mV/s in a 0.1 M KCl solution. Activation cycling was carried out for 20 cycles in 0.1 M KCl. Scan rate dependence measurements were conducted in 0.1 M KCl at scan rates of 10–100 mV/s. The pH stability testing was conducted for 15 cycles in phosphate buffers (KH_2_PO_4_/K_2_HPO_4_) with pH adjusted to 5.4, 6.4, and 7.4. The surface coverage and thickness of the deposited PB were determined by integrating the anodic current response during activation (20th cycle) and dividing this value by the scan rate to obtain the total charge. The surface coverage (in mol/cm^2^) for a 4-electron process and an electrode area of 0.071 cm^2^ was calculated by dividing the obtained charge by (4 × 0.071 cm^2^ × 96,485 C/mol). The average thickness was calculated by dividing the surface coverage by the molar volume of Prussian blue (677 cm^3^/mol).

Analytical characterization (the chronoamperometric detection of hydrogen peroxide and lactate) was conducted with Palmsens EmStat Pico in a three-electrode cell with a double junction Ag/AgCl/3M KCl/3M KCl reference electrode, platinum wire as the counter electrode and the PB-modified inkjet-printed electrode as the working electrode. Hydrogen peroxide detections were run for 30 s at −0.1 V vs. Ag/AgCl/3M KCl/3M KCl reference electrode in 0.1, 0.25, 0.5, 1, 2, 5, and 10 mM of H_2_O_2_ in 0.1 M KCl. Lactate detection was run for 60 s at −0.3 V vs. Ag/AgCl/3M KCl/3M KCl reference electrode in 1, 2, 3, 5, 10, 25, and 50 mM solutions of lactic acid in a phosphate buffer (pH = 6.4) containing 0.1 M KCl, after incubating the sensor in the solution for 1 min. The applied potential of −0.3 V was picked to ensure that the mediator is in its reduced form despite the resistive influences described in the optimization step.

### 2.5. Lactate Oxidase Immobilization

Lactate oxidase was dissolved in phosphate buffer (pH = 7.4) to prepare a 20 mg/mL solution. Next, it was mixed with 1% chitosan in 0.1 M HAc in a volume ratio of 1:1. Three aliquots of 3 µL of the prepared LOx/chitosan solution were drop cast on the PB-modified electrode and left to dry at room temperature for one hour. Each addition was drop cast after the previous layer dried. Finally, 4 µL of PVC (3.3 wt.% solution in THF) was drop cast on top of the assembled biosensor and left to dry for at least three hours before use. All biosensors were used the same day as prepared.

## 3. Results

### 3.1. Prussian Blue Ink Characterization

**Figure 2:**
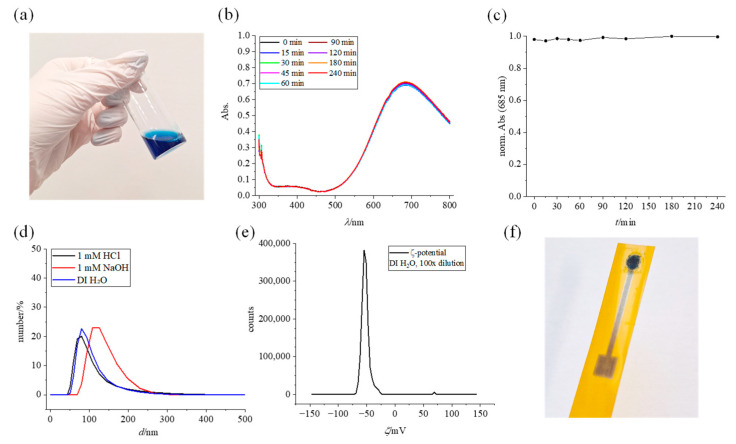
PBNP suspension characterization. (**a**) Photo of a freshly prepared PBNP suspension, (**b**) UV/Vis spectrum of the suspension, (**c**) stability of the suspension during 4 h, (**d**) size distribution obtained by DLS, (**e**) ζ-potential obtained by ELS, and (**f**) fully inkjet-printed graphene–PBNP platform.

The stability of the suspension, as well as the particle size itself, is important for its use in inkjet printing since large and/or precipitated particles would hinder the printing process by clogging the nozzles. The properties of the PBNP suspension were investigated and shown in Figure 2. A freshly prepared suspension (Figure 2a) features a dark blue color that is characteristic of Prussian Blue. A repeatable UV/Vis spectrum (Figure 2b), recorded from the moment the stirrer was turned off and during the next 4 h, shows a broad band at ~700 nm characteristic for the Fe^2+^ to Fe^3+^ charge transfer, with *λ*_max_ = 685 nm. Normalized absorbance of the maxima is depicted in Figure 2c. during four hours and shows no sign of instability of the suspension. Moreover, the suspension was inspected visually and showed no sign of precipitation for at least a week after synthesis.

PBNP suspension was characterized by DLS and ELS to measure size distribution (Figure 2d) and ς-potential (Figure 2e). DLS measurements in pure DI water and 1 mM HCl show overlapping distribution (*Z*_avg_ = 136 nm) and polydispersity index (0.1663 ± 0.0083 for 1 mM HCl and 0.1690 ± 0.0315 for DI water). Measurements conducted in 1 mM NaOH show a greater distribution of larger particles (*Z*_avg_ = 151 nm) with a noticeable drop in PDI during three consecutive repeated measurements, indicative of Prussian Blue instability in alkaline media. ELS measurements were conducted in DI water by diluting the suspension 100 times and yielded a *ς*-potential value of −55 mV, which is in line with a reported value of similarly synthesized nanoparticle suspension [33] and indicates its stability. The conductivity of the suspension was found to be ~0.05341 mS/cm. Rheological and surface properties of the ink were determined in order to evaluate printability and were found to be surface tension 71 mN/m, density 0.997 g/mL, and dynamic viscosity 0.846 mPas. Finally, the PBNP suspension was used for printing on in-house inkjet-printed electrodes, and the fully inkjet-printed platform is shown in Figure 2f. Such platforms were later employed for measurements explained in Section 3.3.

### 3.2. Evaluation of PB Deposition Methods

#### 3.2.1. Glassy Carbon Electrodes

To test the chosen deposition methods and conditions, PB films were first characterized on a glassy carbon electrode. After each deposition procedure, electrodes were dried for 1 h at 100 °C and then characterized via cyclic voltammetry. The first step in characterization was activation (Figure S1a), where successive potential cycling around the reductive system (Prussian White ↔ Prussian Blue) converts the deposited Prussian Blue from insoluble to soluble, more stable form [34,35]. Next, the activated layer is cycled around the same system at different scan rates (10–100 mV/s, Figure S1b). The dependence of peak currents on the square root value of the scan rate is shown in Figure S1c (Randles–Ševčik plot). Finally, the stability of the PB layer was tested in phosphate buffers at different pH and shown in Figure S1d.

Activation of the chemically deposited layers results in the increase in peak currents in the voltammogram, stabilizing after 20 cycles (longer cycling results in diminishing returns). With longer deposition time, the amount of deposited Prussian Blue increases, and the peak currents increase appropriately. Activation has a greater effect on a thicker film, presumably because there is more material to activate, which is evident by a substantially greater increase in the first 10 cycles for 60 min deposition time (Figure S1a). When it comes to scanning rate dependence, it is evident that the thickness of the deposited Prussian Blue layer has a detrimental effect on peak separation. Due to Prussian Blue being an electroactive but nonconductive substance (with resistivity several orders of magnitude higher than that of graphene [36]), a thicker film increases the ohmic resistance, which is noticeable as greater peak separation (Figure S1b) [37]. Randles–Ševčik plots (Figure S1c) are linear for both deposition times tested, indicating a diffusion-controlled process [15]. Finally, pH stability studies (Figure S1d) show that the chemically deposited layers are fairly stable, even in more alkaline media, which is evident by the stability of the peak current, whether in the same solution in time or between solutions of different pH.

The characterization results for drop-cast Prussian Blue nanoparticle (PBNP) suspension are also shown in Figure S1. Here, activation has an opposite effect: successive cycling around the reductive system in 0.1 M KCl slightly reduces the peak currents. This indicates that the drop-cast nanoparticles adhere weakly to the smooth surface of the glassy carbon electrode compared to chemically deposited Prussian Blue, leading to the dissolution of the film. Randles–Ševčik plots show greater linearity compared to chemically deposited films. However, this layer differs significantly when it comes to pH stability; unlike chemically deposited films, which are more strongly adsorbed on the electrode surface (as they were created from a chemical reaction occurring on the surface of the electrode), PBNPs were synthesized separately and then drop cast on the electrode surface, resulting in only mechanical adsorption. Hence, the PBNPs are less strongly bound to the electrode surface and much more susceptible to washing out and dissolution at higher pH. This is evident in Figure S1d, where it is visible that the cathodic peak current drops significantly both within the solution of a certain pH and even more in solutions of increasing pH.

#### 3.2.2. Screen-Printed Carbon Electrodes

The same characterization was conducted on commercial screen-printed carbon electrodes (SPEs). Figure S2 shows the results of the evaluation of the chemically deposited Prussian Blue layer with different deposition times. The result of the chemical deposition method is highly dependent on the substrate [2], as is evident by much smaller peak currents in the activation voltammograms, compared to the GC electrode (Figure S2a). While the activation protocol still increases the peak currents, the effect is much less evident compared to the PB layers formed on the GC electrode. The same effect of peak separation increase with longer deposition times is also apparent, and the Randles–Ševčik plots again show linear dependence, proving the diffusion control of the process. The diffusion process in question is the K^+^ diffusion in and out of the crystal lattice of Prussian Blue [32]. Finally, the electrodes show excellent pH stability, even greater than the one observed on the GCE. This is somewhat expected, thanks to the greater surface area and roughness of the SPE compared to the GCE.

Figure S3 shows the characteristics of PBNP-modified SPE, although, in this step, the PBNP ink was still only drop cast and not printed (which was already explored by Cinti et al. in previous work [27]). As with the drop-cast layer on the GCE, the activation voltammograms confirm the detrimental effect compared to the chemically deposited layer, confirming that the PBNP-modified electrodes do not require this step for stabilization. The shape of the voltammograms is also distinctly different, both on SPE and GCE, compared to the results of the chemical method. The scan rate dependence voltammograms show a slowing increase in current with scan rate, which we assume is the effect of the instability of the drop-cast PBNPs without any additional protection. This effect is also visible in Figure S3c, as the linearity is somewhat disturbed due to nanoparticles washing off. However, the instability of the drop-cast PBNP is again mostly visible in pH stability tests; while stable in acidic media (pH 5.4), the cathodic peak currents drop significantly with pH increase and with more time spent cycling in neutral and alkaline media. The cyclic voltammograms also show a significant reduction both in peak currents and the area of the voltammogram, confirming the loss of the electroactive PB from the surface of the electrode. These results indicate a need for better immobilization of the PBNPs on top of the electrode, which would certainly be the case anyway in a fully assembled biosensor, as the sensing layer would cover and protect the mediator.

The molar surface coverage of the electrodes and the average thickness of the deposited PB was also determined from the activation cyclic voltammograms for all deposition methods. For chemical deposition, the determined surface coverage was 0.54, 1.1, and 1.5 nmol/cm^2^ for 10 min, 20 min, and 60 min deposition, respectively. These values give average thicknesses of 3.6 nm, 7.5 nm, and 10.4 nm, indicating an increase in PB thickness with longer deposition times. The different volumes of PBNP suspension had a more pronounced effect on the surface coverage and thickness. The calculated surface coverage was 1.2, 4.9, and 9.3 nmol/cm^2^ for 10, 20, and 50 µL of suspension, respectively. The respective calculated average thicknesses are 7.8 nm, 33 nm, and 63 nm.

#### 3.2.3. Inkjet-Printed Electrodes

After evaluating the PB deposition methods on the more established commercial electrode configurations, the chemical deposition method and PBNP deposition were employed on in-house inkjet-printed electrodes. The technical drawing and the micrograph of the inkjet-printed graphene electrode are shown in Figure S4, showing consistency between the dimensions given by the model and the final printed electrode. The average diameter of the working electrode is 3.25 mm, and the average edge length of the contact square area is 4.29 mm, yielding an error of 8.3% and 7.3%, respectively. The graphene ink used for electrode fabrication contains ethyl cellulose stabilizer which, while preventing agglomeration, makes the printed electrode nonconductive if not processed after printing.

However, graphene ink is not the only possible contributor to resistance in the system. As observed in Figures S1–S3, as well as in previous work [15,32,37], thicker films of Prussian Blue cause an increase in peak separation as well. Prussian Blue is an electroactive yet nonconductive substance, so the thicker layer causes an increase in ohmic resistance in the system. Inkjet-printed electrodes are also a lot thinner compared to GCE and SPE, having much less conductive material available. While this is beneficial when it comes to reducing costs due to saving material (calculated material costs for a single electrode are less than 20 cents), it makes these electrodes much more susceptible to this kind of resistive influence by modifying them with a nonconductive layer. In this case, it is a matter of compromise between how much of Prussian Blue to deposit and how much of this resistive influence to tolerate. While depositing a thicker layer of Prussian Blue would widen the linear range for the planned lactate detection [8], too much of it significantly increases peak separation in cyclic voltammograms.

**Figure 3:**
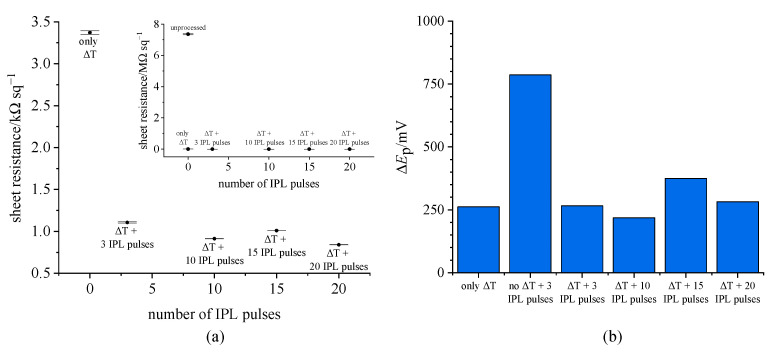
IPL treatment optimization of inkjet-printed graphene electrodes. (**a**) Sheet resistance measurements via four point probe measurements (inset shows the significant drop of sheet resistance of the electrode after treatment compared to the unprocessed electrode); (**b**) peak separation in cyclic voltammograms (PW↔PB) at 10 mV/s recorded on an inkjet-printed electrode chemically modified after corresponding treatment.

We therefore performed optimization of the post-print processing of the inkjet-printed graphene electrodes, with the aim of reducing sheet resistance and peak separation after chemically depositing PB (Figure 3). The inkjet-printed graphene electrodes were processed as follows: after printing, all electrodes were thermally processed as described. Then, a different number of IPL flashes was applied for photothermal treatment (0, 3, 10, 15 and 20 flashes). The sheet resistance values measured by the four-point probe method are shown in Figure 3a (error bars shown for a single electrode processed as described). Even just the thermal post-treatment has an overwhelmingly positive effect on lowering the sheet resistance (Figure 3a inset), from megaohm values of the fully unprocessed electrode (7.36 ± 0.03 MΩ/sq, RSD = 0.41%) down to 3.37 ± 0.02 kΩ/sq (RSD = 0.62%, *n* = 3). However, additional IPL treatment further lowers the resistance to about 1 kΩ/sq (1.11 ± 0.01 kΩ/sq (RSD = 0.65 %, *n* = 3) after 3 pulses; 0.913 ± 0.001 kΩ/sq (RSD = 0.15%, *n* = 3) after 10 pulses; 1.01 ± 0.0002 kΩ/sq (RSD = 0.02%, *n* = 3) after 15 pulses; 0.841 ± 0.0002 kΩ/sq (RSD = 0.02%, *n* = 3) after 20 pulses). While the difference in resistance is not as great between differently flashed electrodes, the difference in the number of IPL flashes used is certainly evident in the cyclic voltammograms. After PB modification, the electrodes underwent cycling from −0.5 to +1.0 V, with results at 10 mV/s summarized in the bar chart in Figure 3b. Evidently, greater post-treatment of the electrodes has an effect on lowering the peak separation, with the lowest values achieved with the electrodes treated with 10 IPL flashes. However, further flashing has an adverse effect on peak separation, and the opposite effect was observed. Moreover, flashing the electrodes with 15 or 20 flashes introduces irreproducibility, completely destroying the surface of some of the electrodes. Figure S5 shows an example of an electrode destroyed after flashing 20 times. The destruction of the electrode with excessive flashing occurs due to the sudden decomposition of the graphene ink stabilizer (ethyl cellulose), which results in gaseous products forming in a very short time period of an applied pulse [38]. Evaporation of the decomposed stabilizers is generally necessary and has a positive effect on the resistance of the inkjet-printed electrode [29], reducing it below 1 kΩ/sq, as well as on reducing the peak separation to a minimal value at 10 flashes in tested conditions. As demonstrated above, further flashing has an adverse effect on the reproducibility of the electrode treatment and results in higher peak separation. Hence, the post-printing processing conditions of 10 IPL flashes after thermal sintering were chosen for further work. The sheet resistance of the electrode characterized in Figure 3a achieved the value of 913 ± 1 Ω/sq (RSD = 0.15%) after applying the optimal treatment parameters. To test interelectrode reproducibility, we measured the sheet resistance of seven different electrodes processed with optimal parameters and obtained the value of 730 ± 69 Ω/sq (RSD = 9.5%, *n* = 7).

Based on the observations above, minimal amounts of PB were deposited on inkjet-printed graphene electrodes for electrochemical testing (i.e., 20 min chemical deposition or 10 µL of PBNP suspension). Nevertheless, these electrodes were not stable in aqueous solutions, possibly due to weak adhesion of the PB layer and the underlying graphene electrode. Since the primary goal of these inkjet-printed platforms is to employ them in enzymatic biosensors with an additional sensing layer with an immobilized enzyme, the PB layer would be protected by the sensing layer anyhow. Moreover, the enzyme itself would have to be immobilized to preserve its activity and enhance its stability [39]. While several enzyme immobilization strategies exist, entrapping the enzyme in a polymeric matrix is advantageous due to the simplicity of the approach and the protection of the enzyme activity compared to other methods like cross-linking or covalent binding [40]. Chitosan is biocompatible and nontoxic to biological systems [41], so it is commonly used in enzymatic biosensor development [9,10,11,16,17,42]. Therefore, it was chosen as the immobilization matrix for our future biosensor assembly. With that in mind, we tested the inkjet-printed electrodes with an added chitosan layer by drop-casting three aliquots of 3 µL of 1% chitosan solution in 0.1 M HAc on top of the PB-modified electrodes. As such, the applied chitosan acts both as protection of the mediator and as the dummy enzyme layer without the enzyme present at this point in testing.

**Figure 4:**
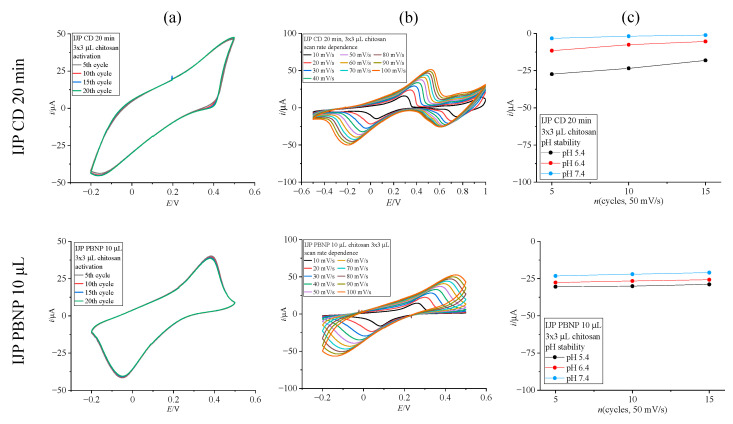
Electrochemical characterization results for optimized deposition methods on inkjet-printed electrodes (top row: chemical deposition 20 min; bottom row: drop-cast PBNP, 10 μL). (**a**) Activation in 0.1 M KCl (scan rate 50 mV/s); (**b**) scan rate dependence recorded in 0.1 M KCl (10–100 mV/s); (**c**) pH stability of deposited films shown as cathodic peak current value extracted from cyclic voltammograms recorded in buffers (pH 5.4, 6.4 and 7.4).

The application of chitosan proved beneficial in keeping the deposited Prussian Blue stable, and the deposited PB layers withheld longer measurement times compared to unprotected layers (Figure 4). As visible in Figure 4a, peak separation is greater for the chemically deposited Prussian Blue layer than for the drop-cast PBNP layer. Therefore, the potential window was widened for scan rate dependence testing in Figure 4b and extends from −0.5 to +1.0 V for the chemically deposited layer. Peak separation values at 10 mV/s are 235.5 mV for Prussian Blue, chemically deposited for 20 min, and 180.5 mV for 10 μL drop-cast PBNP suspension. While the chemically deposited Prussian Blue layer shows greater susceptibility to dissolution in high pH compared to an equivalently prepared layer on commercial electrodes, even with an added protective layer, the drop-cast PBNP layer greatly benefits from the chitosan layer, showing greater stability compared to the equivalently drop-cast NP layer on commercial electrodes with no protection (Figure 4c).

**Figure 5:**
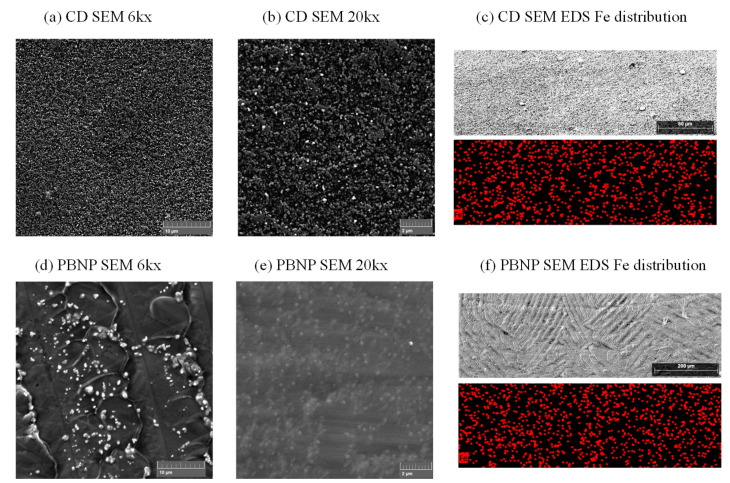
SEM images of optimized PB-modified inkjet-printed electrodes: (**a**–**c**) chemically deposited PB ((**a**)—6k magnification, (**b**)—20k magnification, (**c**)—EDS mapping at lower magnification); (**d**–**f**) drop-cast PB nanoparticles ((**d**)—6k magnification, (**e**)—20k magnification, (**f**)—EDS mapping at lower magnification).

After optimization, FTIR measurement (Figure S6) and SEM imaging (Figure 5) were conducted on IJP electrodes with 20 min chemical deposition, as well as 10 µL PBNP drop-cast suspension. The predominantly graphene electrodes do not exhibit any pronounced peaks, but a slight CN stretch at around 2085 cm^−1^ is visible only on PB-modified electrodes (both types), thus confirming PB formation. SEM imaging shows a nanoparticle-structured PB layer in both chemical deposition (Figure 5a,b) and PBNP modification (Figure 5d,e). Chemically deposited PB shows a uniform deposition of nanoparticles on both magnifications (6k in Figure 5a and 20k in Figure 5b), as well as uniform Fe distribution over a surface scanned at a smaller magnification (Figure 5c). Drop-cast PBNP suspension forms aggregates of nanoparticles visible on the surface at 6k magnification (Figure 5d), while most of the PBNP is trapped beneath a layer of crystallized KCl originating from the suspension (Figure 5e,f). Nevertheless, the EDS mapping shows a uniform Fe distribution over the electrode surface. (Figure 5f).

Finally, the PBNP suspension was printed over the working area of the inkjet-printed graphene electrode. A total of 10 µL was recalculated into a number of layers necessary to cover the working electrode with the equivalent amount of nanoparticle suspension.

### 3.3. Chronoamperometric Detections

#### 3.3.1. H_2_O_2_ Detection on Prussian Blue Modified Inkjet-Printed Electrodes with Chitosan Dummy Layer

**Figure 6:**
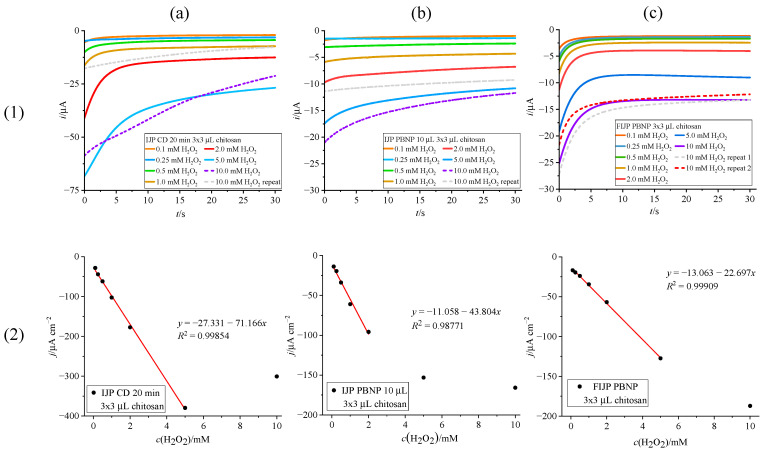
H_2_O_2_ detection with chronoamperometry ((**1**)—chronoamperograms and (**2**)—calibration curves) on electrodes modified by (**a**) 20 min chemical deposition, (**b**) drop casting 10 µL of PBNP suspension, (**c**) fully inkjet printing the PBNP suspension.

Results of chronoamperometric calibration of PB-modified IJP with hydrogen peroxide solutions are shown in Figure 6. All electrodes were tested with a chitosan dummy layer on top, and measurements were conducted at a constant potential set to −0.1 V vs. Ag/AgCl/3M KCl/3M KCl. The chronoamperometric detection is based on the catalytic property of Prussian White to selectively oxidize hydrogen peroxide according to the following reactions [13]:

Fe_4_^III^[Fe^II^(CN)_6_]_3_ + 4e^−^ + 4K^+^ → K_4_Fe_4_^II^[Fe^II^(CN)_6_]_3_

K_4_Fe_4_^II^[Fe^II^(CN)_6_]_3_ + 2 H_2_O_2_ → Fe_4_^III^[Fe^II^(CN)_6_]_3_ + 4OH^−^ + 4K^+^

net reaction: H_2_O_2_ + 2e^−^ → 2OH^−^

Chemically deposited PB film showed the greatest sensitivity of 71.17 µA mM^−1^ cm^−2^, with a linear range of 0.1–5 mM H_2_O_2_ (Figure 6a(2)). However, as visible in Figure 6a(1) from repeated measurements in 10 mM H_2_O_2_ with diminishing currents, the PB layer decomposes rapidly in higher concentrations of peroxide, which is not surprising since the catalytic reduction of H_2_O_2_ produces OH^−^. PBNP suspension drop cast on top of the IJP (Figure 6b) showed better stability in higher concentrations of peroxide (Figure 6b(1)), but as evident in Figure 6b(2), exhibited lower linearity (R^2^ = 0.98771), sensitivity (43.80 µA mM^−1^ cm^−2^) and linear range (0.1–2 mM H_2_O_2_) compared to the chemically deposited PB layer (Figure 6a(2)).

However, the fully inkjet-printed electrode (FIJP) surpassed both chemically and drop-cast modified IJP in terms of linearity (Figure 6c(2)) and stability in higher concentrations of H_2_O_2_ (Figure 6c(1), repeated measurements at 10 mM). While not fully linear up to 10 mM, it showed better reproducibility of current values with repeated measurements at 10 mM. Sensitivity is lower (22.70 µA mM^−1^ cm^−2^) compared to the results shown in Figure 6a,b, but with a trade-off of having a wider linear range (0.1–5 mM), better linearity (R^2^ = 0.99909) and greater stability towards higher concentration of peroxide, these results show great promise for biosensor development.

The analytical performance is comparable to the results found in the literature for hydrogen peroxide determination (Table 1). While screen-printed configurations, even with inkjet-printed Prussian Blue layer, have been explored extensively over the years [4,27,31,32], to our knowledge, this is the first example of a fully inkjet-printed Prussian Blue-modified platform for H_2_O_2_ detection.

#### 3.3.2. Lactate Biosensor Assembly

To test our fully inkjet-printed PB-modified platform with a fully assembled biosensor, lactate oxidase (LOx) was immobilized with chitosan on top of the IJP electrodes. For comparison, both chemical deposition (20 min) and PBNP drop-casting (10 µL) optimized methods were employed. For simplicity, this approach did not use bovine serum albumin (BSA) or any crosslinking methods to immobilize the enzyme. In the fabrication of the lactate biosensor, the chitosan immobilized LOx was covered by an additional layer of PVC, acting both as a protective and a diffusion-limiting layer [10]. The added PVC layer preserved the overall sensing layer from delamination and enabled the testing to be carried out.

As the research for lactate sensing is primarily focused on detecting the lactate levels in a physiological environment, especially for tracking performance in sports and exercise, we tested the assembled lactate biosensors with solutions of lactate in a wide concentration range of 1–50 mM, as the physiological levels of lactate in sweat are considered to be in the range of 3.7–50 mM [43]. Moreover, the lactate standard solutions were prepared in a phosphate buffer solution of pH = 6.4 with 0.1 M KCl. This pH was chosen as a good compromise for lactate oxidase activity, Prussian Blue stability, and the pH value of sweat as the target matrix [43].

**Figure 7:**
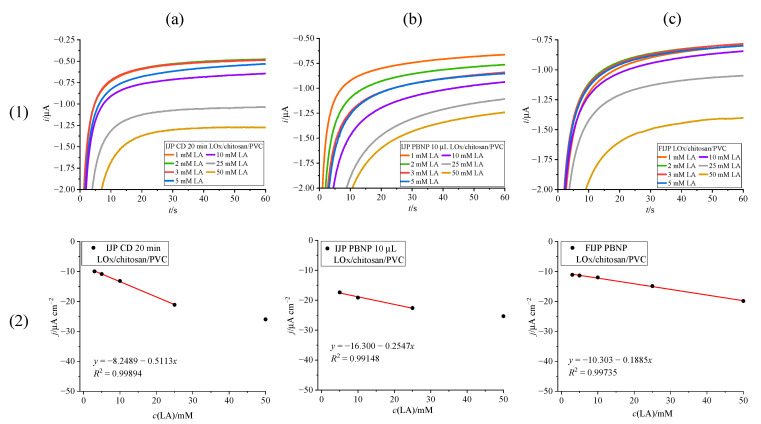
Lactate detection with chronoamperometry on fully assembled inkjet-printed sensor ((**1**)—chronoamperograms and (**2**)—calibration curves for electrodes modified by (**a**) 20 min chemical deposition, (**b**) drop casting 10 µL of PBNP suspension, (**c**) fully inkjet printing the PBNP suspension).

The results of lactate detection are shown in Figure 7. The sensor with chemically deposited Prussian Blue mediator layer (Figure 7a) again shows the best sensitivity (0.5113 µA mM^−1^ cm^−2^), with a linear range of 3–25 mM, partially covering the target window of lactate concentrations, albeit on the lower end. The sensor with the drop-cast PBNP suspension (Figure 7b) exhibited a narrower linear range (5–25 mM) and lower sensitivity (0.2547 µA mM^−1^ cm^−2^) compared to the chemically modified platform. The sensitivity of the fully inkjet-printed platform (0.1885 µA mM^−1^ cm^−2^) is lower than the platform with chemically deposited Prussian Blue or drop-cast PBNP. However, the fully inkjet-printed platform (Figure 7c) showed the best response in terms of linear range (3–50 mM), surpassing the platform modified via chemical deposition and covering the full physiological range of lactate in sweat. These results are promising for facilitated production of low-cost, wearable, and flexible lactate sensors for measuring sweat.

Considering the fact that lactate oxidase is among the less stable oxidase enzymes, which requires more immobilization efforts [39,44], these results are rather in line with the analytical properties of simplified lactate oxidase sensors. Even the use of PVC, while beneficial for widening the linear range of the biosensor, has an adverse effect on the sensitivity. Sensitivity is influenced by the limited diffusion of lactate through the PVC membrane compared to diffusion in the solution [42]. Nevertheless, Table 2 shows a comparison of our prepared biosensors with other lactate sensors found in the literature, and it is evident that our sensors exhibit comparable sensitivity and linear range to presented lactate sensors.

## 4. Conclusions

The chemical deposition and drop casting of nanoparticle suspension were investigated for the deposition of Prussian Blue. Electrochemical activation on commercial glassy carbon and screen-printed electrodes shows the opposite effect on differently deposited Prussian Blue layers, stabilizing the chemically deposited layer while having an adverse effect on the drop-cast nanoparticle layer. The resistive influence of Prussian Blue, while visible on commercial electrodes with an increase in scan rate and amount of deposited Prussian Blue, has a more pronounced effect on the comparatively thinner inkjet-printed graphene substrate. A thorough post-printing treatment is necessary to lower the resistance of the inkjet-printed graphene, as the resistivity of the graphene ink due to stabilizers also affects the electrochemical properties. The optimal post-printing treatment for this platform proved to be thermal processing at 300 °C for 30 min and photothermal treatment via 10 IPL flashes of 539 J.

After optimizing the deposition processes, the PBNP suspension was printed over the working area of the inkjet-printed electrode. The electrodes with optimally deposited Prussian Blue were employed for hydrogen peroxide sensing, and the fully inkjet-printed platform showed better properties (wider linear range, linearity, and greater stability in high concentrations of hydrogen peroxide) compared to the chemically deposited or simply drop-cast Prussian Blue layer, with satisfactory sensitivity (22.70 µA mM^−1^ cm^−2^). Finally, lactate oxidase was immobilized with chitosan and PVC on the optimized PB-modified electrodes. The fully inkjet-printed platform again surpasses the electrodes modified chemically or by drop casting with a very wide linear range (3–50 mM), matching the target window for lactate sensing in sweat. We believe this simple, affordable, and flexible platform is a good starting point for creating fully inkjet-printed biosensors based on Prussian Blue as a mediator.

## Figures and Tables

**Table 1:** Literature overview of partially or fully inkjet-printed (FIJP) hydrogen peroxide sensors (adapted from [23,27]).

Substrate	Electroactive Material	Type	Operating Potential/V	Linear Range/mM	Sensitivity /µA mM^−1^ cm^−2^	Ref.
Graphite SPE	PBNP	SPE with IJP PB	0	0.001–4.5	762	[27]
Carbon SPE	PBNP	SPE with IJP PB	+0.15	0.02–0.7	0.164	[32]
ITO coated glass	PEDOT:PSS/HRP	FIJP	−0.1	0.25–1	0.544	[21]
Polyimide, paper	graphene	FIJP	+0.5	0.2–1.1	3.32	[22]
Paper	graphene PtAuNC@ C6_His16_	FIJP	+0.4	0.05–1	32.3	[23]
PET	SWCNT, SiO_2_ NPs, HRP	FIJP	−0.23	1–3	57	[24]
PVC	AgNP	FIJP	−0.4	0.1–6.8	287	[25]
Paper	MWCNTs, AgNPs	FIJP	−0.3	0.001–0.7	-	[26]
PI/graphene	Prussian Blue (covered with chitosan)	IJP with CD PB	−0.1	0.1–5	71.17	This work
PI/graphene	Prussian Blue (covered with chitosan)	IJP with drop-cast PBNP	−0.1	0.1–2	43.80	This work
PI/graphene	Prussian Blue (covered with chitosan)	FIJP (printed PBNP)	−0.1	0.1–5	22.70	This work

**Table 2:** Literature overview of Prussian Blue-based lactate sensors.

Substrate	PB Deposition Method	Operating Potential/V	Linear Range/mM	Sensitivity ^1^ /µA mM^−1^ cm^−2^	Ref.
SPE carbon	chemical	−0.05	1–50	0.30	[7]
Au	electrodeposition	0	2–30	3.11	[8]
SPE carbon	drop-cast PBNPs	−0.17	1–25	0.031	[9]
SPE carbon/PB	screen printing	−0.1	0–28	1.28	[10]
SPE carbon/PB	screen printing	−0.2	0–30	0.3	[16]
SPE carbon/PB	screen printing	−0.2	0–20	0.35	[17]
IJP graphene	chemical	−0.3	3–25	0.5113	This work
IJP graphene	drop-cast PBNPs	−0.3	5–25	0.2547	This work
IJP graphene	inkjet-printed PBNPs	−0.3	3–50	0.1885	This work

^1^ Calculated from data presented in the article, if not given by the authors in this form.

## Data Availability

Data will be made available from authors upon request.

## Supplementary Materials

The following supporting information can be downloaded at https://www.mdpi.com/article/10.3390/bios15010028/s1. Figure S1: Evaluation of PB films deposited on GC electrode; Figure S2: Evaluation of PB films deposited on commercial carbon SPE via chemical deposition; Figure S3: Evaluation of PB films deposited on commercial carbon SPE by drop casting PBNP dispersion; Figure S4: Technical drawing and dimensions of the inkjet-printed graphene electrode; Figure S5: Micrograph of an inkjet-printed electrode destroyed by excessive IPL flashing; Figure S6: FTIR spectra of PB-modified inkjet-printed electrodes.
