# Supplementary material for: Fully Inkjet-Printed Flexible Graphene–Prussian Blue Platform for Electrochemical Biosensing
Source: Biosensors (Basel). 2025 Jan 8;15(1):28. doi: 10.3390/bios15010028 (PMC11763782; doi:10.3390/bios15010028)
Supplement: Supplementary file 1 [file biosensors-15-00028-s001.zip › biosensors-3372478-supplementary.docx]

**Fully inkjet-printed flexible graphene-Prussian Blue platform for electrochemical biosensing**

Željka Boček, Marko Zubak, Petar Kassal

University of Zagreb, Faculty of Chemical Engineering & Technology, Trg Marka Marulića 19, 10000 Zagreb, Croatia

# Supplementary information


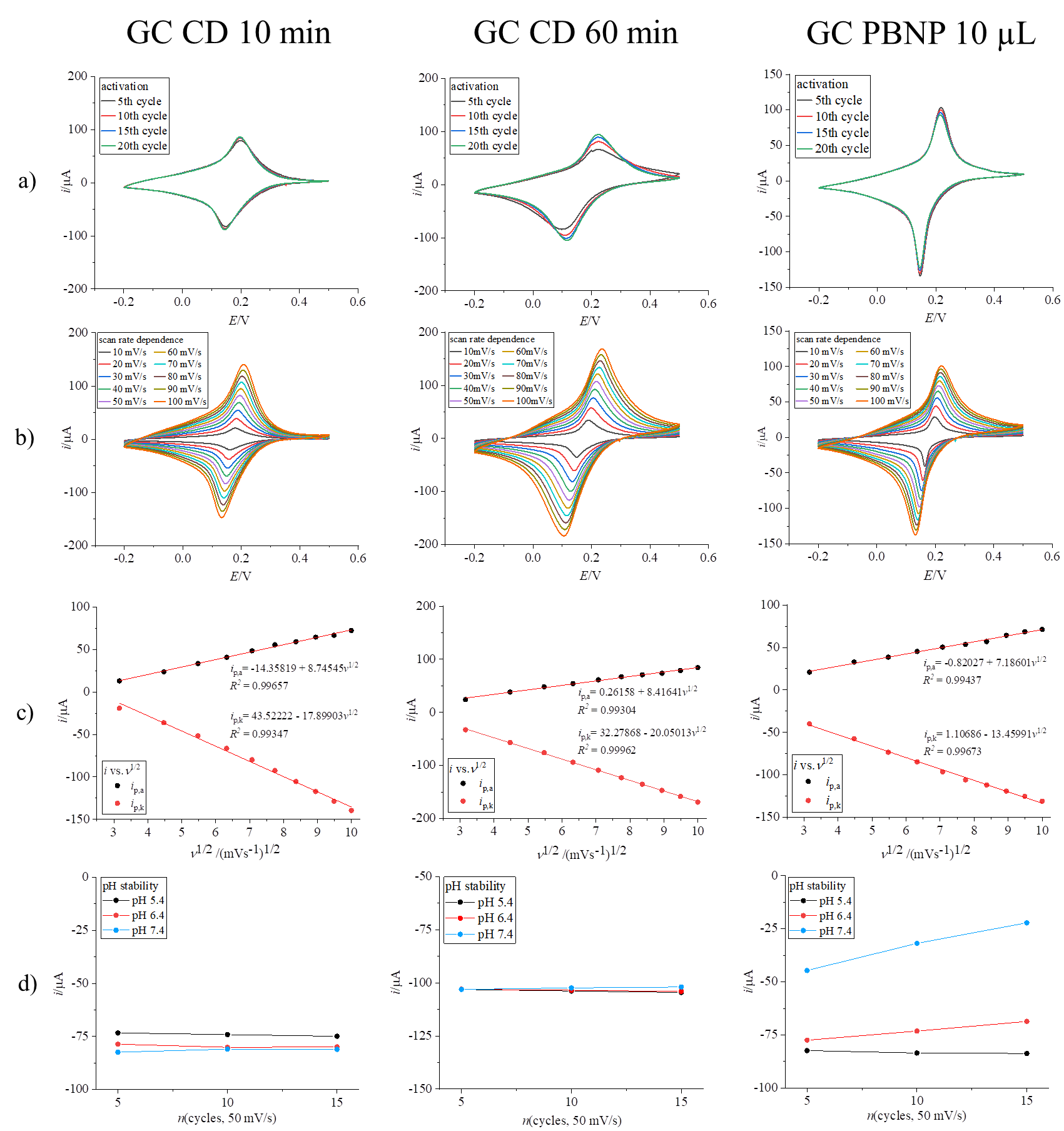


**Figure S1.** Evaluation of PB films deposited on GC electrode via chemical deposition (10 min and 60 min), or by drop casting PBNP dispersion (10 µL). a) Activation in 0.1 M KCl (scan rate 50 mV/s), b) scan rate dependence recorded in 0.1 M KCl (10-100 mV/s), c) Randles-Ševčik plot obtained from voltammograms recorded under c), d) pH stability of deposited films shown as cathodic peak current value extracted from cyclic voltammograms recorded in buffers (pH 5.4, 6.4 and 7.4).


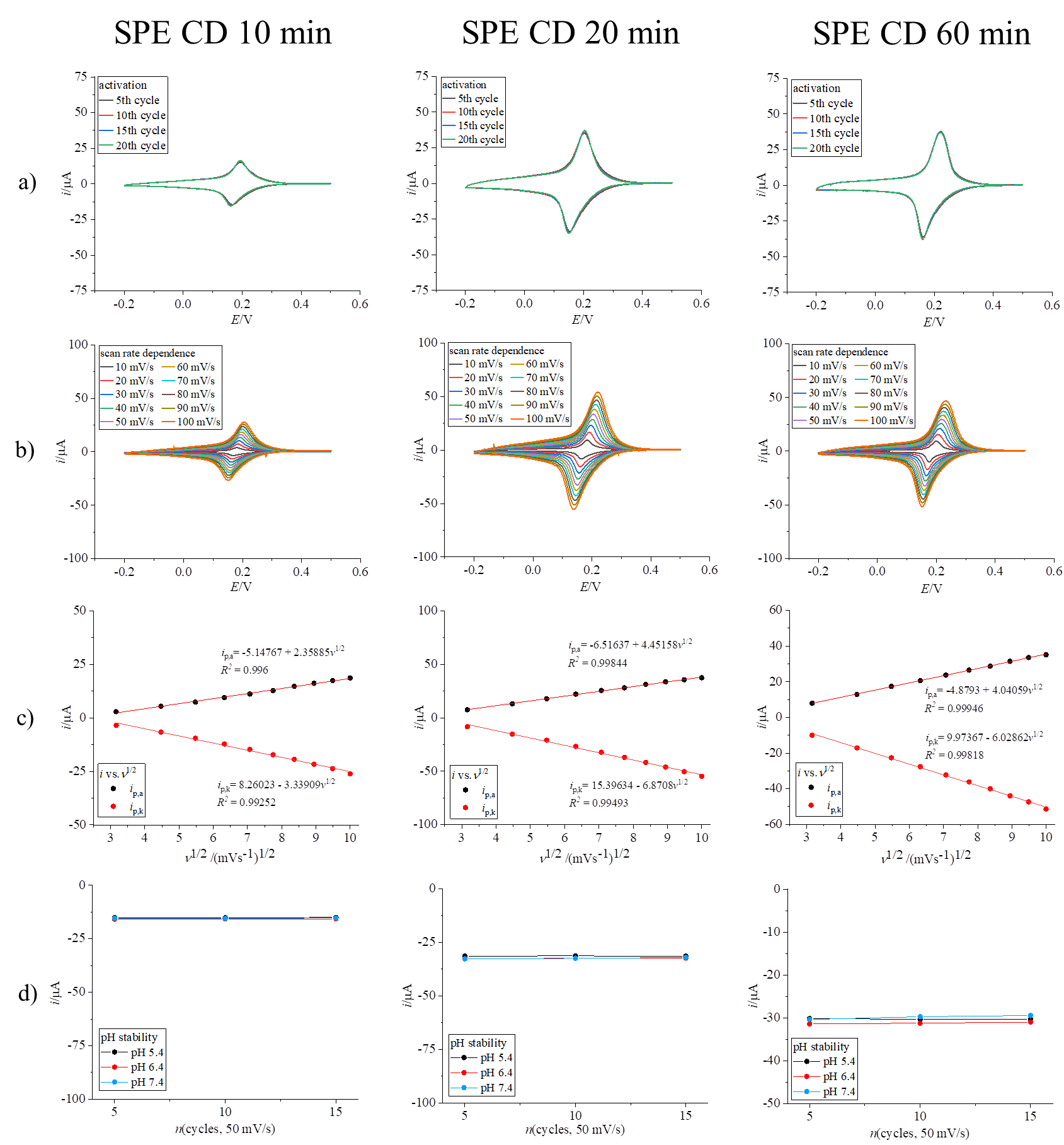


**Figure S2.** Evaluation of PB films deposited on commercial carbon SPE via chemical deposition (1 – 10 min, 2 – 20 min, 3 – 60 min). a) Photo of deposited PB film, b) activation in 0.1 M KCl (scan rate 50 mV/s), c) scan rate dependence recorded in 0.1 M KCl (10-100 mV/s), d) Randles-Ševčik plot obtained from voltammograms recorded under c), e) pH stability of deposited films shown as cathodic peak current value extracted from cyclic voltammograms recorded in buffers (pH 5.4, 6.4 and 7.4).


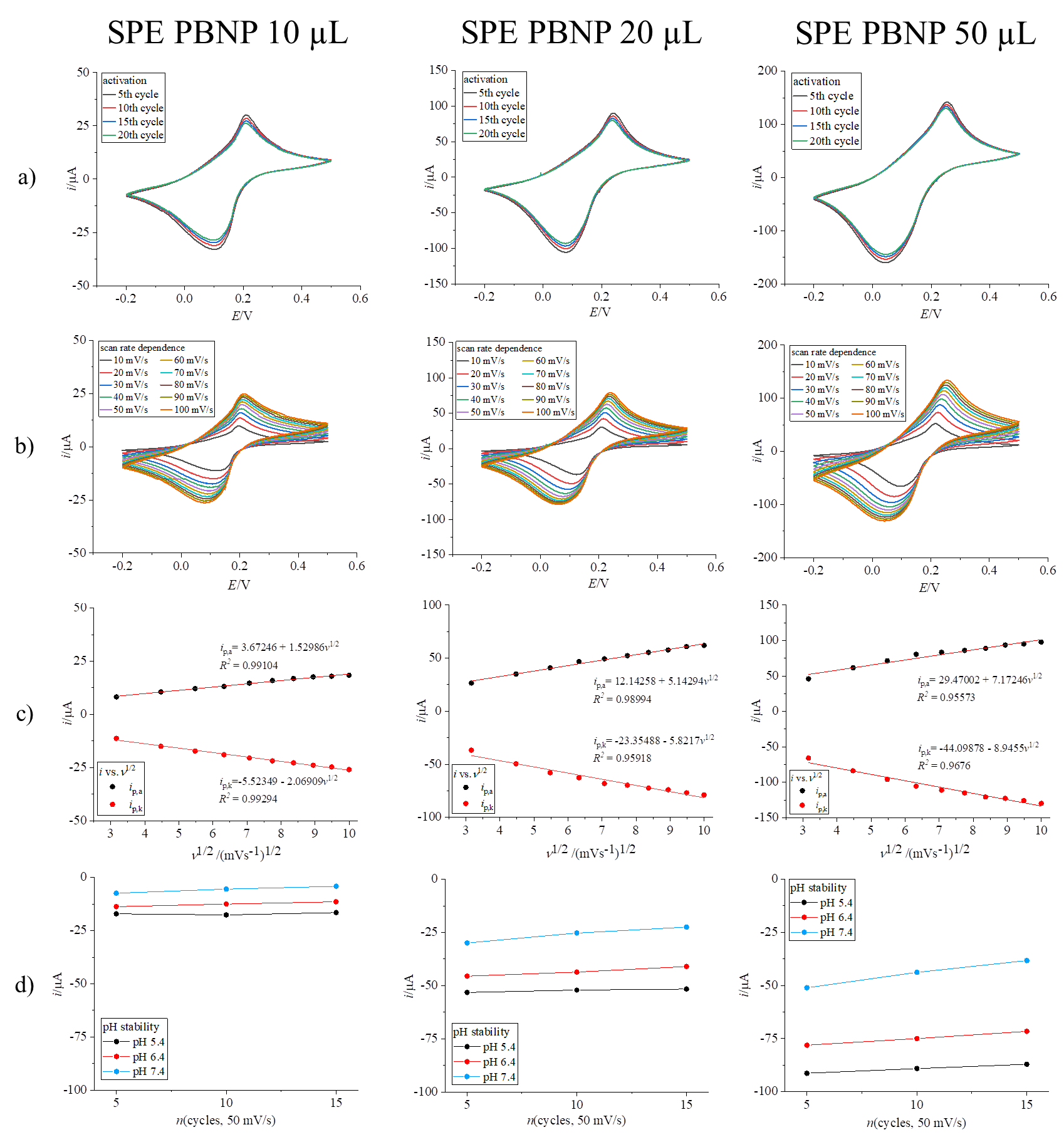


**Figure S3.** Evaluation of PB films deposited on commercial carbon SPE by drop casting PBNP dispersion (1 - 10 µL, 2 – 20 µL, 3 - 50 µL. a) Activation in 0.1 M KCl (scan rate 50 mV/s), b) scan rate dependence recorded in 0.1 M KCl (10-100 mV/s), c) Randles-Ševčik plot obtained from voltammograms recorded under b), d) pH stability of deposited films shown as cathodic peak current value extracted from cyclic voltammograms recorded in buffers (pH 5.4, 6.4 and 7.4).

**Figure S4.** a) Technical drawing of a graphene electrode, b) dimensions of the graphene electrode after inkjet printing.


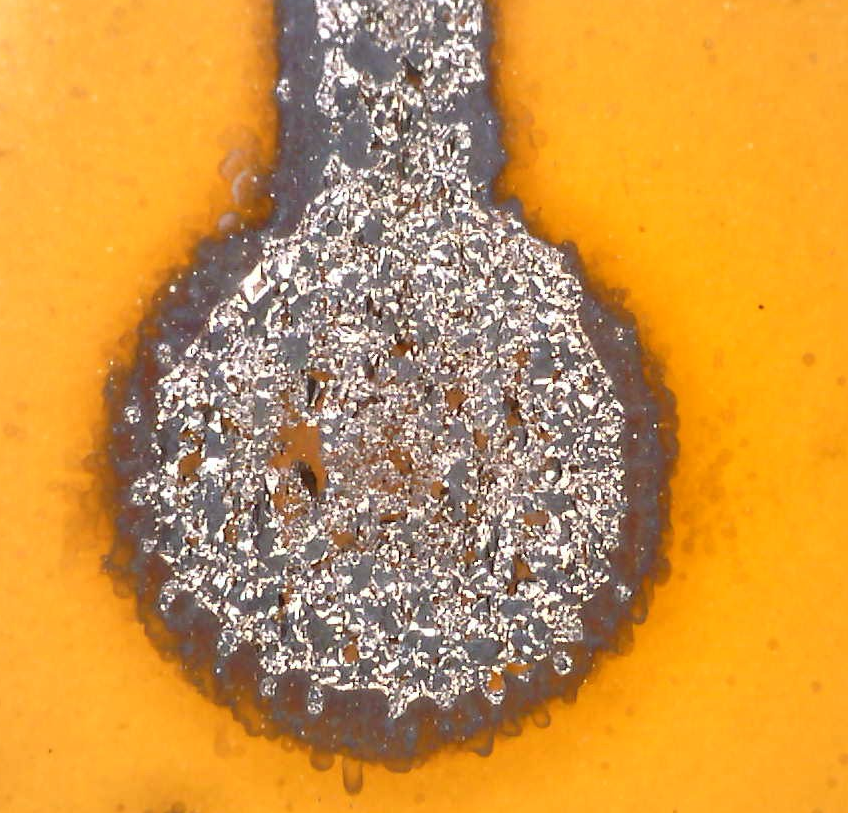


**Figure S5.** Micrograph of an inkjet printed electrode working area destroyed by excessive IPL flashing (20 pulses, *E* = 539 J, *U* = 2500 V).


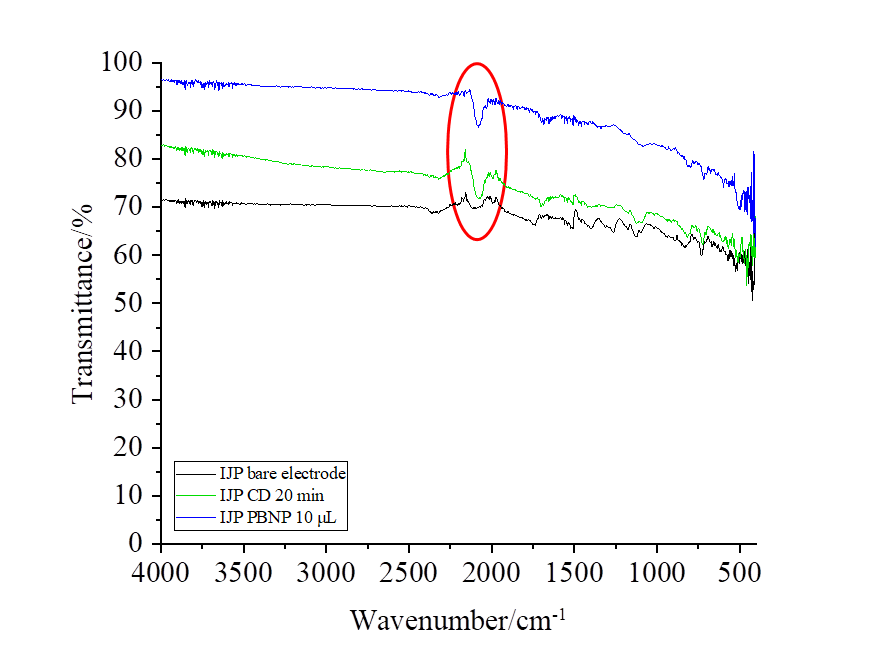


**Figure S6.** FTIR spectra with a CN stretch at around 2085 cm^-1^, visible only on PB modified electrodes, both chemically deposited and with PB nanoparticles.
